# Thyroid disease is a favorable prognostic factor in achieving sustained virologic response in chronic hepatitis C undergoing combination therapy: A nested case control study

**DOI:** 10.1186/1472-6823-11-10

**Published:** 2011-05-24

**Authors:** Huy A Tran, Tracey L Jones, Robert Gibson, Glenn EM Reeves

**Affiliations:** 1 Hunter Area Pathology Service and University of Newcastle, Locked Bag Number 1, Hunter Mail Region Centre, Newcastle, New South Wales 2310, Australia; 2 Hepatitis C Service, Gastroenterology Department, John Hunter Hospital and University of Newcastle, Locked Bag Number 1, Hunter Mail Region Centre, Newcastle, New South Wales 2310, Australia

## Abstract

**Background:**

Interferon-α in combination with ribavirin is the current gold standard for treatment of chronic hepatitis C. It is unknown if the development of autoimmune thyroid disease (TD) during treatment confers an improved chance of achieving sustained virologic response. The aim of this study is to assess the chance of achieving sustained virologic response (SVR) in patients who developed TD during treatment when compared with those who did not.

**Methods:**

We performed a tertiary hospital-based retrospective nested case-control analysis of 19 patients treated for hepatitis C who developed thyroid disease, and 76 controls (matched for age, weight, gender, cirrhosis and aminotransferase levels) who did not develop TD during treatment. Multivariate logistic-regression models were used to compare cases and controls.

**Results:**

The development of TD was associated with a high likelihood of achieving SVR (odds ratio, 6.0; 95% confidence interval, 1.5 to 24.6) for the pooled group containing all genotypes. The likelihood of achieving SVR was increased in individuals with genotype 1 HCV infection who developed TD (odds ratio, 5.2; 95% confidence interval, 1.2 to 22.3), and all genotype 3 patients who developed TD achieved SVR.

**Conclusions:**

Development of TD during treatment for hepatitis C infection is associated with a significantly increased chance of achieving SVR. The pathophysiogical mechanisms for this observation remain to be determined.

**Trial Registration:**

*The Australian New Zealand Clinical Trials Registry (ANZCTR)*: ACTRB12610000830099

## Background

Hepatitis C is one of the major global causes of chronic hepatic infections, particularly in third world countries, and is associated with a significant rate of cirrhosis and hepatoma. In Australia [1,2] and the United States of America [3,4], the burden of disease is significant. Although the incidence has come under control, the associated sequelae unfortunately have been predicted to increase in the coming decades [5]. Consequently, a large and growing number of patients will be expected to undergo treatment for hepatitis C. Of those receiving combination treatment with interferon (IFN)-α and ribavirin (RBV), approximately 5-10% will develop thyroid-related complications [6]. Whilst there are a number of factors in the prediction of favourable hepatic outcome such as genotype, ethnicity, and early viral load reduction [7,8], there are few published reports that assess the development of thyroid disease (TD) in relation to sustained virological response (SVR). Our previous meta-analysis did not find any difference, although this may be due to inherent differences in the published reports [9]. The aim of this study is to investigate the hypothesis that the development of TD in patients treated for HCV is associated with a significantly increased likelihood of attaining SVR.

## Methods

### Patients and control participants

We enrolled 19 patients who developed TD while receiving combination therapy in a major tertiary referral hospital, New South Wales (NSW), Australia. These patients had been assessed by the endocrinology team. All were found to have thyroiditis as previously described [10], except one who developed primary hypothyroidism.

Patients were treated with predetermined IFN-based treatment regimen and thyroid surveillance protocol. Alternative causes of chronic hepatitis were excluded. No patient had dual Hepatitis B and C. All were monitored carefully for protocol adherence and had normal thyroid functions prior to entering treatment. Monthly reviews were performed, including those who developed TD (as defined below) with thyrotropin (TSH) levels, full blood counts, renal and liver function tests as previously described [10]. Thyroid autoantibodies, whilst predictive, were not performed as it did not affect management in the event of TD development.

Because of the scarcity of reported thyroid diseases in relation to SVR in the literature, we performed a nested case control study using a 1:4 ratio of cases to controls. Patients were matched for age, gender, viral load, body weight, alanine and aspartate aminotransferase levels, cirrhotic state and individualised IFN-α2 treatments (either α2a or α2b). The cirrhotic status was determined clinically including ultrasonography and/or computerised tomography.

### Therapy

All patients were treated with combination pegylated IFN-α and RBV therapy. Pegylated IFN- α2a (fixed dose 180 ug) or pegylated IFN-α2b (weight based dose, 1.5 ug/kg of body weight) was injected subcutaneously weekly. Oral RBV was administered twice daily base on the patient's weight (1.0 gm, <75 kg; 1.2 gm, >75 kg) for patients with genotype 1 and the fixed dose (0.8 gm) for patients with non-1 genotype. The duration of treatment depends on the HCV genotypes; genotypes 2 and 3 were treated for 24 weeks and types 1 and 4 for 48 weeks respectively. Treatment was further adjudicated according to viral load results for genotypes 1 and 4 (see below). For genotypes 2 and 3, treatment was continued to the end irrespective of the qualitative viral load at 4 weeks.

### Thyroid disease definition

Thyroid disease was defined as having hypo- or hyper-thyroidism, (clinically and biochemically based). Thyrotoxicosis was defined as having TSH of <0.1 mU/L, fT4 levels >26.0 and/or fT3 levels >6.0 pmol/L respectively.

Hypothyroidism, including subclinical hypothyroidism, was defined as having TSH levels >4.0 mIU/L.

Thyroiditis is defined as the triad of clinical and/or biochemical thyrotoxicosis the current clinical setting, with a reduced/negligible thyroid pertechnetate uptake scan. All uptake scans were reviewed by a specialist nuclear physician consultant. Thyroid autoantibodies may be present but are not considered essential to the diagnosis.

### Virological Response Surveillance Protocol

The following viral load testing protocols were used during the study:

1. Baseline quantitative HCV RNA PCR assays were performed on *all *patients irrespective of genotype.

2. For genotypes 1 and 4, *qualitative *viral load assays were done at weeks 4, 24, 48 and 24 weeks after the completion of therapy. Additional q*uantitative *assay was done at week 12. Treatment was terminated if there was ≤ 2 log_10 _reduction of viral load compared with baseline at the 12^th ^week of therapy or if the qualitative viral load was positive at the 24^th ^week.

3. For genotypes 2 & 3, *qualitative *viral load assay was done at week 4, 24 (at the completion of therapy) and at week 24 follow-up.

SVR was defined as serum HCV-RNA being undetectable by polymerase chain reaction (PCR) at 24 weeks *following *the completion of therapy.

### Statistical Analyses

The study was designed to detect an odds ratio of 2 or more for associations between SVR and thyroid disease development, with 80% power. A 1:4 ratio of cases to controls was used, with significance tests performed at a two-tailed alpha level of 0.05. Odds ratios were adjusted for age, weight, gender and aminotransferase levels. Unconditional multivariate logistic-regression analysis was used to estimated odds ratios and 95% confidence intervals (CIs). Statistical significance was determined using the likelihood-ratio test. Demographic data are presented as percentage and means with standard deviations (SDs). Results were also analysed using a 2 × 2 contingency table with χ^2 ^analysis for comparison of percentages between groups. The Fisher's exact analysis and continuity correction were applied when appropriate. All analyses were performed using Stata 10 software (Stata).

## Results

All subject cases had thyroiditis as previously defined except for one case of primary hypothyroidism and all patients were well matched for all stated criteria, Table 1.

**Table 1:** Characteristics of 95 patients and separate case/control subjects

	All Patients	Cases	Controls	P-values
		TD	No TD	95% CI
	N = 95	N = 19	N = 76	
*Demographic factors*				
Female gender - number (%)	55 (57.9%)	11 (57.9%)	44 (57.9%)	NSS
Mean age (years) ± SD	47 ± 8	47 ± 8	48 ± 7	NSS
Weight (kg) ± SD	69.7 ± 10.1	70.6 ± 8.5	69.8 ± 10.7	NSS

*Hepatic factors*				
Cirrhosis - number (%)	5 (5.3%)	1 (5.3%)	4 (5.3%)	NSS
ALT (<45 U/L) ± SD	54 ± 24	56 ± 26	53 ± 25	NSS
AST (1-30 U/L) ± SD	39 ± 11	41 ± 12	38 ± 12	NSS

*Virologic factors*				
Genotypes - number (%)				
• 1	55 (58.0%)	11 (58.0%)	44 (58.0%)	NSS
• 3	40 (42.0%)	8 (42.0%)	32 (42.0%)	NSS
Viral Load				
- At baseline (log IU/mL) ± SD	6.44 ± 0.52	6.22 ± 0.56	6.64 ± 0.53	NSS
*Treatment regimens - number (%)*				
IFN-α2a	55 (57.9%)	11 (57.9%)	44 (57.9%)	NSS
*together with*				
Ribavirin				
1.0 gm (patients ≤ 75 kg)	20 (36.4%)	4 (36.4%)	16 (36.4%)	
1.2 gm (patients >75 kg)	15 (27.3%)	3 (27.3%)	12 (27.3%)	
0.8 gm	20 (36.4%)	4 (36.4%)	16 (36.4%)	
IFN-α2b	40 (42.1%)	8 (42.1%)	32 (42.1%)	NSS
*together with*				
Ribavirin				
1.0 gm (patients ≤ 75 kg)	10 (25.0%)	2 (25.0%)	8 (25.0%)	
1.2 gm (patients >75 kg)	10 (25.0%)	2 (25.0%)	8 (25.0%)	
0.8 gm	20 (50.0%)	4 (50.0%)	16 (50.0%)	

Results are expressed as Means ± Standard Deviations (SDs). CI, Confidence Intervals; IFN, Interferon; NSS, Non Statistically Significant; ALT, Alanine Aminotransferase, AST; Aspartate Aminotransferase.

Sustained virologic response was observed in 84.2% of patients who developed TD compared with 53.9% in those who did not. The difference between proportions was significant at 0.30 (95% Confidence Interval (CI), 0.10 - 0.50, p < 0.05).

Overall, univariate logistic regression analysis revealed that cases which developed TD, had a 6.0 times greater likelihood of achieving SVR compared with controls (95% confidence interval (CI), 1.5 to 24.6). The likelihood of achieving SVR was even greater for genotype 1 cases with TD versus those without TD (OR, 5.2; 95% CI, 1.2 to 22.3), and all genotype 3 patients who developed TD also achieved SVR, Table 2.

**Table 2:** Sustained Virologic Responses (SVR), Odds Ratios (OR) and 95% Confidence Intervals (CI) analyses for *all *patients, genotype 1, 3 and regimen-specific subgroups

*GENOTYPE 1 ( N = 55)*								
**Treatment regimens**	Pegylated IFN-α2a and RBV 1000 mg		Pegylated IFN-α2a and RBV 1200 mg		Pegylated IFN-α2b and RBV 1000 mg		Pegylated IFN-α2b and RBV 1200 mg	
	Control 16	TD 4	Control 12	TD 3	Control 8	TD 2	Control 8	TD 2
EVR*	Not applicable (see text)							
**ETR - number (%)**	9 (56.3%)	3 (75.0%)	9 (75.0%)	3 (100.0%)	4 (50.0%)	2 (100.0%)	5 (62.5%)	2 (100.0%)
**SVR - number (%)**	8 (50.0%)	3 (75.0%)	7 (58.3%)	2 (66.6%)	4 (50.0%)	2 (100.0%)	5 (62.5%)	2 (100.0%)

***GENOTYPE 3 (N = 40)***								

**Treatment regimens**	Pegylated IFN-α2a and RBV 800 mg				Pegylated IFN-α2b and RBV 800 mg			
	Control N = 16		TD N = 4		Control N = 16		TD N = 4	
EVR*	Not applicable (see text)							
**ETR - number (%)**	**16** **(100.0%)**		**4** **(100.0%)**		**16** **(100.0%)**		**4** **(100.0%)**	
**SVR - number (%)**	16 (100.0%)		4 (100.0%)		16 (100.0%)		4 (100.0%)	
**SVR for genotype 1: Odds Ratio, 95% CI**	5.2, 1.2 to 22.3							
**SVR for genotype 3**	Control (n = 32) 100.0%				TD (n = 8 ) 100.0%			
	All 40 cases achieved SVR making the combination of Genotype 3 and TD a perfect predictor of SVR in this group.							

**Treatment related SVR IFN-α2a vs IFNα2b**	Non Statistically Significant							

**OVERALL SVR: ODDS RATIO, 95% CI**	**6.0, 1.5 to 24.6**							

Multivariate logistic regression analysis showed that presence of TD and genotype was the only two variables significantly associated with SVR. Modelling with these two independent variables revealed that individuals who developed TD had a 6 folds greater likelihood of achieving SVR compared with controls (95% confidence interval (CI), 1.5 to 24.6).

## Discussion

The prevalence of hepatitis C is now controlled in the Western world [5] but continues to increase in third world and developing countries due to lack of needle exchange program and medical infrastructure. In Australia, the numbers have stabilized and more recently declined [2]. The current established and accepted treatment for chronic hepatitis C infection is the combination of pegylated IFN-α and RBV. This treatment regimen delivers a cure rate of approximately 50% of cases [11]. The favourable factors in response to treatment include genotypes (2 and 3 genotypes have better response), compliance, duration of treatment and early viral load reduction [8]. Additional positive prognosticators consist of lower body mass index, lack of co-existing liver disease and ethnicity (Asians and Caucasians have a higher SVR than African Americans) [7]. The relationship between TD and SVR had been scarcely addressed and in our solitary meta-analysis, the outcome was negative [9].

Hypothetically, when treatment is delivered, the immune system is stimulated sufficiently in an attempt to eradicate the virus and in the process may also be intense enough to trigger TD, as an unintended consequence. Because the presence of TD may signify a supercharged and heightened immune response which in turn increases the chance of eradicating the virus. Studies have shown that in patients who developed TD, immune marker levels such as Interleukin-6 (IL-6), Tumor Necrosis Factor-alpha (TNF-α) and chemokines are relatively higher and thus in the process may potentially be more effective at achieving SVR [12]. Furthermore, the presence of thyroid hormones *in vitro *also potentiates the antiviral action of IFN in cultured human cells [13]. This hypothesis was observed in our previous report in which all 11 IFN-related thyroid cases achieved SVR [10]. However, a subsequent meta-analysis failed to find any relationship although this might be due to inherent differences within the various reports [9]. This additional nested case control study in which the cases were matched in a ratio of 1 case to 4 controls finds further supportive evidence that the development of TD in HCV patients whilst undergoing combination with RBV and IFN-α treatment is a favorable prognostic factor for achieving SVR. It is reassuring that other factors are matched including age, gender, weight, viral load, cirrhosis and transaminase levels; some of which can influence final SVR status. It is also interesting that there was one primary hypothyroid case which was clearly not the hypothyroid phase of the thyroiditis. In this case, the patient responded well to routine thyroxine supplement.

The viral kinetic responses bear little relationship to the development of TD. It is not possible to assess the relationship between the early viral response (EVR), its associated viral load and the timing of TD development. This is because TD tends to develop approximately at the 18^th ^week of treatment [10]. Furthermore, the critical end-point of this report is the assessment of SVR, irrespective of other landmark viral load studies during treatment. However, end of treatment responses (ETR) appear to confirm previous observation that it is often a reliable predictor of SVR. Most patients who achieved ETR also completed SVR except for the two genotype 1 patients who relapsed as defined by failure to achieve SVR. There was a 6-fold increase in the chance of achieving SVR with TD development for the pooled group containing all genotypes (odds ratio, 6.0; 95% confidence interval, 1.5 to 24.6). The likelihood of achieving SVR was increased in individuals with genotype 1 HCV infection who developed TD (odds ratio, 5.2; 95% confidence interval, 1.2 to 22.3). All genotype 3 patients who developed TD achieved SVR. Although our control cohort has a SVR rate of 100%, the overall reported and accepted rate approximates 80%, perhaps suggesting an additional synergistic effect in this genotype subgroup. Because of this overall high response rate and in order to quantify this amplified increment, there would need to be a much larger cohort. This is unlikely to be achievable given the low incidence of true TD occurring in this setting.

Whilst the thyroid gland is clearly implicated in this study, it remains undetermined whether thyroiditis is a mediator or the result of processes leading to SVR. While the development of a more effective immune response to HCV may unmask underlying thyroid autoimmune tendencies, it is also possible that acute exposure to supraphysiological concentrations of thyroid hormones (THs) may confer favourable immunomodulating activities leading to SVR. It is not yet known if the behaviour of the thyroid conditions in this setting is any different from those arising de novo and hence provides differing influences on the final hepatitic viral status.

HCV infection results in the induction of IFN-α and -β production as part of the innate immune response [14], with subsequent activation of natural killer cells, maturation and proliferation of dendritic cells, proliferation of memory T cells, and prevention of T-cell apoptosis [15]. These effects are important in the mediation of a heightened tendency toward organ-specific autoimmunity (including TD) in patients with HCV. The addition of exogenous IFN-α potentiates the risk of inflammatory autoimmunity in an already vulnerable and primed thyroid gland.

The exposure to the two different forms of IFN-α may be a potential confounder in eliciting SVR. These two IFNs differ significantly in their pegylation characteristics which may translate into their pharmacokinetics and biological activity. Recent reports indicated that there was a slightly higher SVR rate with pegylated IFN-α2a compared with α2b [16]. However, recent practice guidelines did not distinguish between the two in their recommendations [17-19]. There appears to be insufficient data to guide preference of one over the other. Similarly, our findings do not indicate any difference in SVR with regards to the two types of IFN-α, but again this is due to the small numbers.

The second potential influencing factor is the exposure to THs. Normal levels are unlikely to be effective as all other euthyroid and treated patients would otherwise have normal circulating levels. However, the presence of THs *in vitro *also potentiates the antiviral action of IFN in cultured human cells (13). In the presence of IFN, THs can enhance the immunomodulation such as HLA-DR antigen expression (15). It is therefore possible but highly speculative that the favorable outcome seen in these patients is related to the exposure to supraphysiological concentrations of THs. However, it is equally possible that THs are not involved but is a mere para-phenomenon in a vigorous and exaggerated response to IFN therapy.

Finally, the recent discovery of the association between single nucleotide polymorphisms (SNPs) near the interleukin (IL) 28B gene locus showed that this plays an important part in the spontaneous recovery, response to treatment and attaining SVR from HCV infection [20-23]. This SNPs may reflect differential levels of the immunomodulating cytokine IFN-λ-3, but the possibility that individuals at higher risk of TD development harbor favorable SNPs remains to be explored [24].

One of the shortcomings of this report is the small sample size, due primarily to the low prevalence of the condition. This was a major determinant of the choice of study design, with nested case-control studies offering important data on relatively uncommon end-points with little potential for additional bias. Nevertheless, collaboration with prominent centers would potentially allow prospective designs to be pursued, hence enhancing the power of the study; such studies would require standardization of thyroid surveying and definitions of thyroid disease. Secondly, there may be different genetic or regional differences as other reports, albeit retrospective, failed to find such a consistent pattern of SVR amongst TD cases [25-27]. The sampling of both cases and controls from the same patient cohort reduced the chance of selection bias.

### Clinical implications

The finding from this study tentatively raises the question of whether thyroxine might be useful as an adjuvant addition to the treatment armamentarium. It is critical that this finding is confirmed (or refuted) by larger and independent studies because recent data suggested that in HCV cases with SVR, cirrhosis and its associated morbidities can be reversible [28].

## Conclusions

This nested case control study strongly indicates that TD is associated with a higher chance of achieving SVR, especially in genotype 1. This association mandates larger prospective multicentre trials to conclusively address this hypothesis if thyroxine is to be considered a potential adjuvant to current treatment regimen.

## Competing interests

The authors declare that they have no competing interests.

## Authors' contributions

HAT conceived the study, participated in its design, assisted with data collection and statistical analysis, and coordinated and helped to draft the manuscript. GEMR contributed to the statistical and meta-analytical methods, and participated in the discussion and drafting of the manuscript. TLJ, RG gathered, provided the data, and participated in the discussion and drafting of the manuscript. All authors read and approved the final revised manuscript.

## Pre-publication history

The pre-publication history for this paper can be accessed here:

http://www.biomedcentral.com/1472-6823/11/10/prepub
